# Assessment of cognitive functions in individuals with systemic lupus erythematosus

**DOI:** 10.1186/s42358-025-00461-8

**Published:** 2025-11-03

**Authors:** Juliana Rosa Pires Vieira, Andrea Toledo de Oliveira Rezende, Danilo Rocha Dias, Nilzio Antonio da Silva, Marcos Rassi Fernandes

**Affiliations:** 1 Scrictu Senso, Program in Health Sciences, School of Medicine, FMUFG, Goiânia, Goiás, Brasil; 2 Department of Restorative Dentistry, School of Dentistry, UFMG, Uberlândia, Minas Gerais, Brazil; 3 Rheumatology and Emeritus, Scrictu Senso, Program in Health Sciences, School of Medicine, FMUFG, Goiânia, Goiás, Brasil; 4 Health Sciences, Scrictu senso, Program in Health Sciences – School of Medicine – FMUFG, Goiânia, Goiás, Brasil

**Keywords:** Systemic lupus erythematosus, Cognition, Rey’s complex figure, Neuropsychological test

## Abstract

**Introduction:**

Neurocognitive changes may hinder the autonomy and independence of patients diagnosed with Systemic Lupus Erythematosus (SLE), being considered as one of the main negative outcomes.

**Objectives:**

To evaluate the cognitive functions of patients diagnosed with active SLE and in remission at the Rheumatology Outpatient Clinic of the Hospital das Clínicas, School of Medicine, Universidade Federal de Goiás.

**Methodology:**

This was a cross-sectional study, non-probabilistic sample and consecutive type. MiniMental State Examination (MMSE), Five Digits Test (FDT), and Rey’s Complex Figures (FCR) were used in 83 patients in the period from November 2021 to May 2022. Patients with depressive disorders and users of alcohol and other drugs were excluded.

**Results:**

The assessed cognitive functions - attention, memory, language, and executive functions - showed mild impairments, corroborating previous studies. There was a significant difference (p > 0,05) concerning cognitive performance when comparing patients with active SLE and in remission groups. Cognitive function was not associated with time of diagnosis.

**Conclusion:**

In this sense, cognitive dysfunctions were present in patients with active SLE and remission. The results showed mild cognitive impairment in patients diagnosed with SLE.

## Introduction

Systemic Lupus Erythematosus (SLE) is a chronic, multisystem inflammatory disease of unknown cause and autoimmune nature [1], which is more common in women [2]. The prevalence in Brazil is 2 to 5 cases per 100,000 inhabitants [3], with a clinical picture marked by activity and remissions, causing alterations in the nervous and peripheral systems [4].

Neuropsychiatric manifestations affect between 21 and 95% of individuals with SLE [4], and the American College of Rheumatology (ACR) [5] has described 19 neuropsychiatric syndromes in SLE, including cognitive dysfunction [5]. These alterations compromise functions such as attention, memory, language, and executive functions [6], and there are validated instruments that are considered the “gold standard” [7] for measuring them, including the Mini-Mental State Examination (MMSE) [8, 9, 10], the Five Digit Test (FDT) [11] and Rey’s Complex Figures (FCR) [12].

The prevalence of cognitive dysfunction in SLE has ranged from 3 to 81% among studies [13], with around 10% severely impacting functional outcomes, work, and quality of life, hindering activities of daily living, autonomy, and independence for these individuals [14]. Although cognitive dysfunction negatively affects individuals with SLE, no studies investigated this damage using these three instruments in the context of SLE. In this sense, this study aimed to evaluate and compare the neurocognitive functions of individuals with active SLE and SLE in remission and verify the association between SLE activity, and neurocognitive dysfunctions.

## Method

### Study design and location

This cross-sectional study was conducted at the Rheumatology Outpatient Clinic of the Hospital das Clínicas of the Federal University of Goiás between November 2021 and May 2022.

### Sampling

The study sample was non-probabilistic, consecutive, and consisted of individuals with active SLE or remission. Individuals with active SLE were classified according to SLEDAI 2 K *(Systemic Lupus Erythematosus Disease Activity Index 2000)* with a score ≥ 6, while SLE was in remission when the score was zero [15].

### Eligibility criteria

The inclusion criteria were (a) age ≥ 18 years; (b) a diagnosis of SLE, according to the criteria of the American College of Rheumatology and the *European Alliance of Associations for Rheumatology* - ACR/EULAR [15] and (c) schooling ≥ 4 years;

The exclusion criteria were (a) individuals with psychiatric (depressive) or neurological disorders and (b) individuals who used alcohol or other drugs [16]. All the patients answered the ASSIST questionnaire, which is the one defined by the World Health Organization and the Ministry of Health (Brazil) as the standard [16]. After analyzing each patient’s answers, if there had been “alcohol use” in the last three months, the patient would be excluded. The existing comorbidities of the individuals who participated in the study were checked, and those diagnosed with other rheumatological diseases were not included.

### Data collection

Initially, the Alcohol, Cigarette, and Other Substances Involvement Screening Test (ASSIST) was applied to verify the use/abuse of alcohol and other drugs [16], followed by the Depressive Thoughts Scale (DTS) [17] to ascertain the possibility of including the individual in the study. In the event of negative results, the MMSE, FDT, and FCR instruments were applied in sequence, at a single moment, in the form of an interview.

### Instruments used

The MMSE is a screening instrument that covers global cognitive function (G factor), such as time and space orientation, memory, attention, language, and executive function [8, 9]. The proposed cut-off points are 20 points for illiterates, 25 points for people with 1 to 4 years of schooling, 26.5 for 5 to 8 years, 28 points for those with 9 to 11 years, and 29 points for more than 11 years, which totals a maximum score of 30 points [9]. In this sense, for factor G, a “lower” score, lower than those mentioned above, is considered to be mild cognitive impairment, and an “average” score is defined as no impairment [10].

The FDT assesses cognitive processing speed, attention subtypes, automatization and progressive task learning, verbal fluency, inhibitory control, and cognitive flexibility. The FDT is divided into (a) Simple, measuring processes such as Reading and Counting and (b) Complex, measuring more elaborate mental processes such as Active Mental Control. Test performance; classified according to their performance in exceptionally low (below the 5th percentile), low (below the 25th percentile), average (25–75th percentile), high (above the 75th percentile) and exceptionally high (above the 95th percentile) performers [11].

The FCR assesses perceptual activity, executive functions, and visual memory. The total score for both copying and memory is 36 points. The total score, after correction, is converted into a percentile and distributed into five categories: a) 10 to 20: below average; b) 25 to 40: lower average; c) 50: average; d) 60 to 70: upper average and e) 75 to 100: above average [12].

### Outcome

The study’s primary outcome was the evaluation of the cognitive domains assessed in the MMSE, FDT, and FCR instruments described above, demonstrating the cognitive function of individuals with SLE at the time of application.

### Independent variables

The independent variables were age (in years), gender (male/female), schooling (Elementary school (> seven years), Secondary school (up to 12 years) and Higher education (>16 years)), marital status (married, single, divorced, widowed), profession (formal employment/informal employment), treatment adherence (In a pre-established questionnaire, each patient answered about their treatment adherence, marking () yes, () no or () sometimes, regarding the use of medication), and time since diagnosis (up to 10 years/ ≥ 10 years). The main manifestations of disease activity were arthritis, malar rash, proteinuria, and complement alterations.

### Data analysis

The data was analyzed using descriptive statistics, bivariate tests, and association tests (chi-square test, Fisher’s exact test, and Mann-Whitney test) to verify the dependence between the variables related to the clinical characteristics and neuropsychological profile of the participants. The association between time of diagnosis, SLE activity, and cognitive function was tested using Spearman’s correlation test. The reliability of the MMSE, FDT, and FCR instruments was checked using Cronbach’s alpha coefficient. The Shapiro-Wilk test was used to analyze the normal distribution of the quantitative variables. All statistical analyses were done using the Statistical Package for the Social Sciences software (IBM-SPSS 19.0). The significance level adopted was 5%.

### Ethical aspects

The HC/UFG Research Ethics Committee approved the study under CAAE No. 52,926,321.0.0000.5078.

## Results

From the total of 131 individuals with SLE who entered the Rheumatology Outpatient Clinic, 48 were excluded due to neuropsychiatric disorders (38 due to Depressive Disorders and 10 due to alcohol and other drug use). Eighty-three participants were included in the study, 29 of whom were active and 54 in remission (Table 1). The mean time of diagnosis was 11.64 years (standard deviation 7.53).

**Table 1:** Sociodemographic and clinical characteristics of individuals throughout the table active Systemic Lupus Erythematosus (SLE) and in remission

Sociodemographic characteristics	SLE active n = 29	SLE remission n = 54	***p-value***
**Age**^**a**^	43 (16,83)	38,31 (13,26)	0,278
**Sex **^**b**^	**n (%)**	**n (%)**	0,652
Male	2 (6,9)	4 (7,4)	
Female	27 (93,1)	50 (92,6)	
**Education**^**c**^			0,628
Elementary school (>7 years)	10 (34,5)	13 (24,1)	
High school (up to 12 years)	13 (44,8)	28 (51,8)	
Higher education (> 16 years)	6 (20,7)	13 (24,1)	
**Occupation**^**c**^			0,044^*^
With occupancy	11 (37,9))	33 (61,1)	
No occupation	18 (62,1)	21 (38,9)	
**Clinical Characteristics**	M (SD)	M (SD)	
**Diagnosis time**^**a**^	14,45 (6,97)	10,13 (7,45)	0,005^*^
Diagnostic time ^c^			0,004^*^
< 10 years	8 (27,6)	33 (61,1)	
≥ 10 years	21 (72,4)	21 (38,9)	
**Adherence to treatment**^**b**^	**n (%)**	**n (%)**	0,018^*^
Adherence	8 (27,6)	31 (57,4)	
Partial adherence	14 (48,3)	18 (33,3)	
No adherence	7 (24,1)	5 (9,3)	

^a^ Mann-Whitney test

^b^ Fisher’s exact test

^c^ Chi-square test

^*^ Significant for p < 0.05

Data expressed in mean (standard deviation) or n (%)

When evaluating the Cronbach’s Alpha results of the instruments used, all showed reliability (Minimental = 0.75, Simple FDT = 0.86, FDT Complex = 0.86, FCR Copy = 0.79, FCR Memory = 0.79).

Regarding the cognitive function assessed in the MMSE, of the 83 individuals who took part in the study, 68.67% had a “lower” level with scores ranging from 20 to 28 points, considered mild cognitive impairment. There was a significant association between the MMSE result (68.67% exceptionally low and 31.32% low/average) and SLE activity (*p = 0.*04), with 82.8% of individuals with active SLE having mild cognitive impairment. In contrast, among individuals with SLE in remission, this condition was observed in 61.1%. Considering the Simple FDT, Complex FDT, FCR Copy, and FCR Memory tests, most participants had a “exceptionally low” level, and there was no relation between the neurocognitive function assessed by these tests and SLE activity (Table 2).

**Table 2:** Neurocognitive function test results are distributed between individuals throughout the table active SLE and SLE in remission

	Total SLE n = 83	Active SLE n = 29	SLE remission n = 54	p-value
	n (%)	n (%)	n (%)	
Cognitive Function (MiniMental) ^a^				0,04^*^
Exceptionally low	57 (68,67)	24 (82,8)	33 (61,1)	
Low/average	26 (31,32)	5 (17,2)	21 (38,9)	
Simple FDT^a^				0,06
Exceptionally low	58 (69,87)	24 (82,8)	34 (63)	
Low/average	25 (30,12)	5 (17,2)	20 (37)	
FDT Complex^a^				0,72
Exceptionally low	63 (75,90)	22 (75,9)	39 (72,2)	
Low/average	22 (26,50)	7 (24,1)	15 (27,8)	
FCR Copy^a^				0,16
Exceptionally low	62 (74,69)	24 (82,8)	38 (70,4)	
Low/average	19 (22,89)	4 (13,8)	15 (27,8)	
FCR Memory^b^				0,12
Exceptionally low	76 (91,56)	27 (93,1)	49 (90,7)	
Low/average	5 (6,02)	0 (0)	5 (9,3)	

^a^ Chi-square test

^b^ Fisher’s exact test

^*^ Significant for p < 0.05

## Discussion

The results of our study showed that in the total SLE group, as well as in active SLE and SLE in remission, the individuals obtained a “exceptionally low” level in the MMSE test, demonstrating mild cognitive impairment.

This corroborates studies [18, 19, 20, 21], which confirmed alterations in various cognitive domains, including executive functions (attention and processing speed, visuospatial processes, working memory, cognitive flexibility, and inhibitory control), language and memory (learning) in individuals with SLE, observing significant differences compared to healthy individuals. Langesee et al. used the *CNS Vital Signs Neurocognitive Testing Report* test. They concluded that the cognitive performance of individuals with active SLE or in remission was significantly low in the psychomotor speed, motor speed, and focused attention domains than healthy controls [18].

Zarfeshani et al. compared healthy individuals with SLE and found a higher frequency of cognitive alterations, with deficits in executive functions (sequential processing) and memory in the latter [15]. Monahan et al. explained that the very activity of SLE can lead to inflammation of the Central Nervous System, resulting in impairment related to cognitive dysfunction [19].

Tektonidou et al. observed that ischemic lesions caused by antiphospholipid antibodies in patients diagnosed with SLE lead to increased blood-brain barrier permeability, intrathecal autoantibodies, and other inflammatory mediators involved in cognitive dysfunction [22]. In addition, Oláh et al. mentioned that the use of medications with neurotoxic and psychoactive effects and other manifestations as a result of SLE, such as stroke, seizures, depression, or anxiety, could also lead to cognitive dysfunction [23].

Concerning the FDT and FCR tests, there was no significant association between SLE activity and neurocognitive alteration. Monahan et al., who used FCR to assess cognitive dysfunction, also did not identify proportional frequencies in cognitive functions between those with active SLE and those in remission [19]. No studies analyzed the FTD test to assess cognitive dysfunction and SLE.

In this study, when comparing neurocognitive functions in individuals with SLE activity, the results of the MMSE showed a significant association between the two, and the prevalence of individuals with mild cognitive impairment was higher in those with active SLE. Moore et al. also found a positive association between disease activity and cognitive dysfunction [24]. Hanly et al. reported that excessive production of the pro-inflammatory cytokine interleukin-6 (IL-6) could lead to an exacerbated immune response and, consequently, greater disease activity, which has been associated with cognitive dysfunction [25]. Trapero et al. described that IL-6 is a cytokine that induces the inflammatory expression of acute phase proteins, and these act as large pyrogens, increasing vascular permeability, lymphocyte activation, and antibody synthesis, and this contributes to cognitive symptoms that include the main clinical signs of dementia and cognitive impairment [26].

Yue et al. compared the time of diagnosis in individuals with SLE between those with and without cognitive impairment. They found that those with CD had an average of 68 months of disease diagnosis while those without CD had 36 months [27]. Hanly et al. also found that individuals diagnosed with SLE more than five years previously had significantly greater cognitive impairment than those with a shorter diagnosis time [28].

Mohanan et al. described potential aspects throughout the disease that can influence the development of cognitive domains, including disease activity [19]. Utz et al. explained that SLE can cause progressive and irreversible brain damage, such as neuronal loss, demyelination, and changes in brain structure and function, which can trigger cognitive dysfunction [29]. Another reason mentioned by Tektonidou et al. was that the comorbidity of antiphospholipid antibody syndrome is common in individuals with SLE and associated with cognitive behavior [22].

Our study has limitations since data collection was carried out in just one center in the public health network of a university hospital, in addition to the possibility of selection bias due to non-probabilistic sampling of an outpatient clinic specializing in rheumatology.

On the other hand, we obtained a sample of individuals with SLE, all of whom underwent a standardized assessment of cognition, including gold-standard tests (MMSE, FDT, and FCR) applied together. Another consideration was the inclusion of specific instruments to measure the use and abuse of alcohol and other drugs and the Depressive Thoughts Scale to exclude mimicking factors concerning the outcomes.

## Conclusion

The results of the MMSE showed mild cognitive impairment in individuals diagnosed with SLE. In this test, there was a significant association with SLE activity, with a higher prevalence of individuals with mild cognitive impairment among those with active SLE. However, there was no significant association between SLE activity and the results of the FDT and FCR.

## Data Availability

The data sets used and/or analyzed during this study are available from the corresponding author upon reasonable request.

## Abbreviations

SLE: Systemic Lupus Erythematosus
ACR: American College of Rheumatology
CD: Cognitive dysfunction
MMSE: MiniMental State Examination
FDT: Five Digit Test
FCR: Rey’s Complex Figures
SLEDAI 2K: Systemic Lupus Erythematosus Disease Activity Index 2000
ACR/EULAR: American College of Rheumatology/European Alliance of Associations for Rheumatology
ASSIST: Alcohol, Cigarette, and Other Substances Involvement Screening Test
DTS: Depressive Thoughts Scale
CNS: Vital Signs Neurocognitive Testing Report test
